# Combined Effects of Romosozumab and Zoledronate on the Development of Osteonecrosis of the Jaw

**DOI:** 10.1111/odi.70331

**Published:** 2026-04-06

**Authors:** Hae‐Seo Park, Shihyun Kim, Seo‐Yeong An, Reuben H. Kim, Jongho Choi, Moon‐Young Kim

**Affiliations:** ^1^ Department of Oral and Maxillofacial Surgery, College of Dentistry Dankook University Cheonan Republic of Korea; ^2^ Department of Oral Pathology, College of Dentistry Gangneung‐Wonju National University Gangneung‐si Republic of Korea; ^3^ The Shapiro Family Laboratory of Viral Oncology and Aging Research Los Angeles California USA; ^4^ UCLA Jonsson Comprehensive Cancer Centre Los Angeles California USA

**Keywords:** healing, MRONJ, osteoclast, romosozumab, zoledronate

## Abstract

**Objective:**

This study aimed to assess the occurrence of medication‐related osteonecrosis of the jaw (MRONJ) in mice following the sequential administration of romosozumab and zoledronate and to confirm their inhibitory effects on osteoclast differentiation in vitro.

**Materials and Methods:**

Thirty‐five female C57BL/6 mice were divided into three groups: romosozumab followed by zoledronate (ROM+ ZOL), saline followed by zoledronate (ZOL), and control (Con) group receiving saline. After drug administration, the maxillary first molars were extracted. Bone healing and necrosis were evaluated using micro‐computed tomography, histomorphometry, and immunohistochemistry. To investigate the mechanism underlying the in vivo study results, the effects of romosozumab and zoledronate were evaluated by analysing tartrate‐resistant acid phosphatase staining and actin ring formation after differentiation of RAW 264.7 cells.

**Results:**

The ROM+ZOL group exhibited more severe bone necrosis than the ZOL and Con groups. The ROM+ZOL group demonstrated a lower bone volume fraction and a reduction in the number of sclerostin‐positive cells, suggesting enhanced osteonecrosis when romosozumab was administered before zoledronate. Moreover, both zoledronate and romosozumab effectively suppressed osteoclast differentiation and function in vitro.

**Conclusions:**

Sequential administration of romosozumab followed by zoledronate may exacerbate the risk of MRONJ, underscoring the need for careful consideration in clinical applications.

## 1 Introduction

Bisphosphonates are widely used bone resorption inhibitors that suppress osteoclast activity (Rogers et al. 2000). They are commonly prescribed for the treatment and management of conditions such as osteoporosis, metastatic cancer, and metabolic bone diseases (Hampson and Fogelman 2012). Since the initial reports by Marx (2003) and Ruggiero et al. (2004) on bisphosphonate‐related osteonecrosis of the jaw (BRONJ), extensive research has been conducted to understand its aetiology, prevalence, and treatment strategies. It has become evident over time that osteonecrosis can also occur with medications other than bisphosphonates, such as denosumab (Taylor et al. 2010; Aghaloo et al. 2010), tyrosine kinase inhibitors including imatinib (Nicolatou‐Galitis et al. 2013), and angiogenesis inhibitors such as bevacizumab (Estilo et al. 2008). Due to this expanded list of drugs causing osteonecrosis, the American Association of Oral and Maxillofacial Surgeons recommended replacing the term BRONJ with medication‐related osteonecrosis of the jaw (MRONJ) in 2014 (Ruggiero et al. 2014). In 2022, romosozumab was added to the list of drugs associated with MRONJ (Ruggiero et al. 2022).

Romosozumab is an anti‐sclerostin monoclonal antibody with dual actions: it stimulates bone formation and inhibits bone resorption (Bandeira et al. 2017). By inhibiting sclerostin, romosozumab activates the Wnt signalling pathway, which leads to enhanced osteoblast differentiation and activity. This stimulates bone formation and increases osteoprotegerin (OPG) levels, thus indirectly inhibiting osteoclast activity and reducing bone resorption (Lim and Bolster 2017). Clinical trials have demonstrated the efficacy of romosozumab in patients with osteoporosis. For example, Cosman et al. (2016) reported that romosozumab reduced the incidence of vertebral fractures compared with placebo in postmenopausal women, and Saag et al. (2017) found that the combination of romosozumab and alendronate significantly reduced the frequency of vertebral and hip fractures compared with alendronate alone. Based on these findings, the Endocrine Society recommends romosozumab as a first‐line treatment for osteoporosis in patients with a T‐score below −2.5 and multiple fractures (Shoback et al. 2020). However, MRONJ has been reported as a side effect in patients treated with romosozumab (Cosman et al. 2016; Saag et al. 2017; Palla et al. 2023).

Animal models have been widely used to study MRONJ. Since the introduction of a rat MRONJ model by Sonis et al. (2009), various MRONJ models have been developed using rodents (Yan et al. 2022). These models generally induced MRONJ by modifying systemic and local risk factors such as drug type, treatment duration, and administration route, as well as by performing dental extractions (Williams et al. 2014) or inducing infections such as pulpitis or periodontitis (Song et al. 2016; Williams et al. 2020). Among these, bisphosphonates such as zoledronate are often used either alone (Soma et al. 2021) or in combination with dexamethasone (Jang et al. 2015), whereas receptor activator of nuclear factor‐κB ligand (RANKL) inhibitors are sometimes combined with agents such as cyclophosphamide (Hayano et al. 2020). However, no studies have yet examined MRONJ using a bone formation inducer such as romosozumab, despite its increasing clinical use in osteoporosis treatment.

In this study, we aimed to investigate the occurrence of MRONJ in mice following tooth extraction after the sequential administration of romosozumab and zoledronate and confirm the inhibitory effects of zoledronate and romosozumab on osteoclast differentiation in vitro. These findings were expected to provide insights into whether this treatment regimen induces MRONJ and the potential risks involved.

## 2 Materials and Methods

### 2.1 In Vivo Analysis

#### 2.1.1 Experimental Animals

Thirty‐five 8‐week‐old female C57BL/6 mice were used in this study. After a 1‐week acclimatization period, the mice were divided into experimental groups. The animal facility was maintained at an average temperature of 22°C, a relative humidity of 50%, and a 12‐h light–dark cycle. Water and feed were provided *ad libitum* throughout the experimental period. The study protocol was approved by the Dankook University Institutional Animal Care and Use Committee (DKU‐23‐004), and all procedures adhered to the relevant regulations for animal experimentation.

#### 2.1.2 Drug Administration

The mice were divided into three groups: ROM+ZOL group (*n* = 15), which received romosozumab (Evenity, Amgen, Thousand Oaks, CA, USA) followed by zoledronate (Zoledronic Acid inj., Daewoong, Seoul, Korea); ZOL group (*n* = 15), which received saline followed by zoledronate; and Con group (*n* = 5), which received saline only. Romosozumab or saline was administered subcutaneously at 10 mg/kg once a week for 3 weeks. Following this, both the ROM+ZOL and ZOL groups received zoledronate intravenously at 125 μg/kg twice a week for 8 weeks. After drug administration, maxillary first molar extractions were performed. The respective drug regimens were continued for an additional 3 weeks after extraction, after which the animals were sacrificed (Figure 1).

**FIGURE 1:**
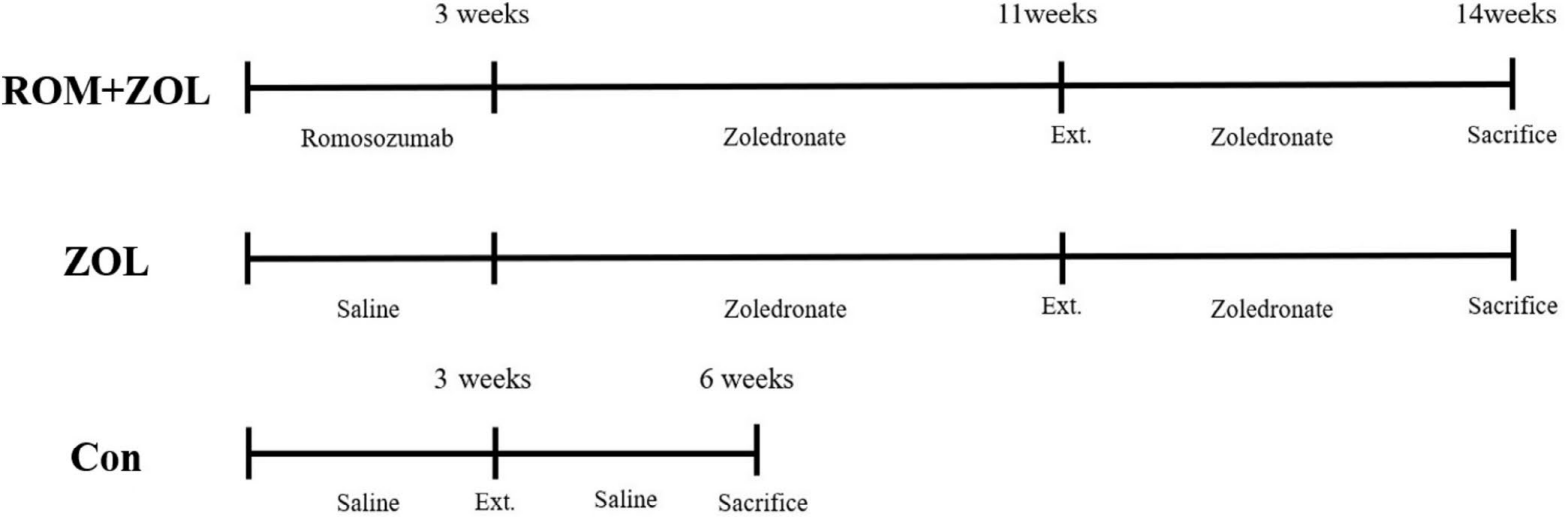
Experimental process diagram.

#### 2.1.3 Anaesthesia and Tooth Extraction

Anaesthesia was induced via intraperitoneal injection of zoletil (Zoletil 50, Virbac, Carros, France) at 30 mg/kg and xylazine (Rompun, Bayer Korea, Seoul, Korea) at 10 mg/kg. Tooth extractions were performed using a dental explorer, and meloxicam (Metacam 2%, Boehringer Ingelheim Korea, Seoul, Korea) was administered subcutaneously at 1 mg/kg for postoperative analgesia.

#### 2.1.4 Tissue Preparation and Clinical Analysis

Following tooth extraction, drug administration was continued for 3 weeks before the mice were euthanised via CO_2_ asphyxiation. The maxillae were collected for analysis, and clinical photographs of the extraction sites were taken to document healing outcomes. Tissues were fixed in 10% neutral‐buffered formalin for subsequent histological and imaging analyses. All in vivo analyses, including micro‐computed tomography (micro‐CT), histomorphometric, and immunohistochemical analyses, were performed at 3 weeks after tooth extraction.

#### 2.1.5 Micro‐CT and Morphometric Analysis

Tissue samples were scanned using a SKYscan 1176 micro‐CT system (Bruker, Kontich, Belgium) at 65 kV and 385 μA with an Al 1 mm filter. Three‐dimensional images were reconstructed with a voxel size of 9 μm using the computed tomography reconstruction program (NRecon, Bruker, Kontich, Belgium) and analysed with an image analysis software (CTAn, Dataviewer; Bruker, Kontich, Belgium). The extraction site was designated as the region of interest (ROI) in the axial view of micro‐CT scans. This ROI was defined to follow the natural contour of the extraction socket, including the interradicular septum, and extended from the alveolar bone crest to the most apical extent of the socket. The registered grey‐value images were segmented into binary images and a fixed threshold (lower and the upper grey threshold values of 70 and 255 respectively). Samples that exhibited root fractures were excluded from the quantitative analysis. Tissue volume (TV) and bone volume (BV) were quantified, and the bone volume fraction (BV/TV) was subsequently calculated for each sample.

#### 2.1.6 Histomorphometric Analysis

##### 2.1.6.1 Haematoxylin and Eosin (H&E) Staining

Following euthanasia, the maxillae of the mice were fixed in 10% neutral‐buffered formalin and decalcified in 5% ethylenediaminetetraacetic acid (EDTA; pH 7.4) at 4°C for 6 weeks. The samples were then dehydrated, embedded in paraffin, and sectioned at a thickness of 5 μm. H&E staining was performed, and the sections were examined under a light microscope. For all histomorphometric and immunohistochemical analyses, the ROI was defined horizontally from the outer surface of the buccal plate across the extraction socket, encompassing the palatal wall and the immediately adjacent palatal bone, and vertically from the alveolar bone crest to the palatal bone. The number of empty lacunae and areas with five or more contiguous empty lacunae (indicative of necrotic bone) were measured. The number of empty lacunae per square millimetre and the percentage of necrotic bone area were calculated for each specimen.

##### 2.1.6.2 Tartrate‐Resistant Acid Phosphatase (TRAP) Staining

Deparaffinised sections were treated with a solution of 50% acetone and 50% ethanol for 10 min and incubated with TRAP staining solution (Takara, Tokyo, Japan) at 37°C. The TRAP solution contained naphthol AS‐BI phosphate and a fast red‐violet LB diazonium salt mixture. Sections were counterstained with toluidine blue. Osteoclasts (defined as cells with ≥ 3 nuclei) were counted, and the number of TRAP‐positive cells per square millimetre in the ROI was calculated.

#### 2.1.7 Immunohistochemical Analysis

Maxillary tissues were fixed in 10% neutral‐buffered formalin and decalcified in 5% EDTA (pH 7.4) for 6 weeks. Tissue sections (5 μm) were deparaffinised, and heat‐mediated antigen retrieval was performed using citrate buffer (pH 6.0). Endogenous peroxidase activity was blocked with 3% hydrogen peroxide, and non‐specific binding was blocked using 2% bovine serum albumin. Primary antibodies targeting osteoprotegerin (OPG, ab124820), RANKL (ab216484), and sclerostin (SOST, AF1589) were applied. After incubation with primary antibodies, the sections were treated with ImmPRESS polymer reagents and visualised using DAB chromogen. The sections were then counterstained with H&E and examined under a light microscope. Negative controls included rabbit immunoglobulin G isotype antibodies and omission of the primary antibody. The percentage of RANKL and OPG‐positive areas, the RANKL/OPG ratio, and the number of SOST‐positive cells per square millimetre were calculated. All micro‐CT, histomorphometric and immunohistochemical analyses were performed by a single blinded observer and were repeated twice at least 2 weeks apart. Intra‐observer reliability was evaluated using the intraclass correlation coefficient (ICC), and the mean of the two readings was used for statistical analyses.

### 2.2 In Vitro Cell Analysis

#### 2.2.1 Cell Culture and Treatments

RAW 264.7 cells were obtained from the Korean Cell Line Bank (Seoul, Korea) and cultured in high‐glucose Dulbecco's Modified Eagle's Medium (Invitrogen, Carlsbad, CA, USA) supplemented with 10% foetal bovine serum and 1% penicillin/streptomycin. A total of 5 × 10^5^ of RAW 264.7 cells were maintained in a humidified incubator at 37°C with 5% CO_2_ and sub‐cultured every 2 days. Osteoclasts originate from the haematopoietic lineage of monocytes or macrophages and absorb and degrade bone matrix (Boyle et al. 2003). For osteoclast differentiation with RAW 264.7 cell line, the cells were transferred to alpha‐Minimum Essential Medium with nucleosides (Thermo Fisher Scientific, Waltham, MA, USA), supplemented with 20 ng/mL macrophage colony‐stimulating factor (M‐CSF; R&D Systems, Minneapolis, MN, USA) and 50 ng/mL soluble RANKL (sRANKL, Oriental Yeast Co. Ltd., Tokyo, Japan), and incubated for 2 days. For stimulation experiments, RAW 264.7 cells were treated with 25 μg/mL zoledronate and 6.45 mg/mL romosozumab.

#### 2.2.2 Western Blotting

Total protein was extracted from RAW 264.7 cells using 1× radioimmunoprecipitation assay buffer (Sigma‐Aldrich, St. Louis, MO, USA) supplemented with a protease inhibitor cocktail (Roche Diagnostics, Basel, Switzerland). The extracted proteins were incubated at 95°C for 5 min with 1× sodium dodecyl sulphate (SDS) loading buffer and then loaded onto 10% SDS‐polyacrylamide gels for electrophoresis. Following separation, proteins were transferred onto nitrocellulose membranes.

The membranes were blocked in 5% bovine serum albumin prepared in phosphate‐buffered saline with Tween for 30 min and then incubated at room temperature for 3 h with primary antibodies against RANK (Santa Cruz Biotechnology, CA, USA, 1:1000 dilution), NFATc1 (Santa Cruz Biotechnology, 1:1000 dilution), and cathepsin K (Santa Cruz Biotechnology, 1:1000 dilution). Glyceraldehyde‐3‐phosphate dehydrogenase (AB Frontier, Seoul, Korea, 1:3000 dilution) was used as a loading control. After washing, the membranes were incubated for 1 h with horseradish peroxidase‐conjugated anti‐mouse and anti‐rabbit immunoglobulin G secondary antibodies (Cell Signaling Technology, Danvers, MA, USA, 1:5000 dilution). Protein bands were detected using a chemiluminescent substrate (Millipore, St. Louis, MO, USA) and visualised with the FUSION Solo S imaging system (Vilber, Eberhardzell, Germany). All experiments were repeated at least twice. Quantitative analysis of protein expression was performed using ImageJ (National Institutes of Health, Bethesda, MD, USA).

#### 2.2.3 Actin Ring Formation

After treatment with an M‐CSF and sRANKL mixture, the RAW 264.7 cells were exposed to zoledronate and romosozumab and cultured on coverslips for 3 days. The cells were then gently rinsed with Dulbecco's phosphate‐buffered saline (DPBS) and fixed with 4% paraformaldehyde for 20 min. Following fixation, the cells were incubated with Alexa Fluor 488 Phalloidin (Cell Signaling Technology; 1:100 dilution) at room temperature for 30 min to visualise the actin cytoskeleton.

After another gentle rinse with DPBS, the cells were mounted using Fluoroshield Mounting medium with 4′,6‐diamidino‐2‐phenylindole (Abcam, Waltham, MA, USA) for nuclear staining. Images were acquired using a STELLARIS 5 confocal microscope (20× objective; Leica Microsystems, Wetzlar, Germany). For quantitative analysis, images were randomly captured from seven different fields per group, and actin ring formation was assessed by counting cells with more than three nuclei.

#### 2.2.4 TRAP Staining

For TRAP staining, 3 × 10^3^ of RAW 264.7 cells were cultured on coverslips and fixed with 4% paraformaldehyde for 20 min. The cells were then permeabilised with 0.5% Triton X‐100 for 20 min. After three washes with DPBS, the samples were stained using the TRAP Stain Kit (Solarbio Science & Technology Co. Ltd., Beijing, China) according to the manufacturer's instructions. Staining was conducted at 37°C in the dark.

Following staining, the slides were washed with DPBS, mounted with Dako Fluorescence Mounting Medium, and examined under an inverted light microscope equipped with an Olympus U‐TV camera (Olympus, Tokyo, Japan).

### 2.3 Statistical Analysis

All data are expressed as mean ± standard error of the mean (SEM). For in vivo analysis, data exhibiting a normal distribution were compared using one‐way analysis of variance (ANOVA) followed by Tukey's honestly significant difference (HSD) post hoc test. For data that did not follow a normal distribution, the Kruskal‐Wallis test was performed followed by the Dunn's post hoc test. For in vitro analysis, one‐way ANOVA was conducted, followed by Tukey's HSD post hoc test for pairwise comparisons. A *p*‐value < 0.05 was considered statistically significant. All experiments were performed at least in triplicate.

## 3 Results

### 3.1 In Vivo Analysis

#### 3.1.1 Morphometric Analysis by Micro‐CT

The BV/TV ratio at the extraction sites was 69.35% ± 5.26% in the ROM+ZOL group, 68.85% ± 4.48% in the ZOL group, and 83.81% ± 2.21% in the Con group. There was no significant difference between the ROM+ZOL and ZOL groups; however, both groups showed significantly lower BV/TV ratios than the Con group (*p* < 0.05; Figure 2).

**FIGURE 2:**
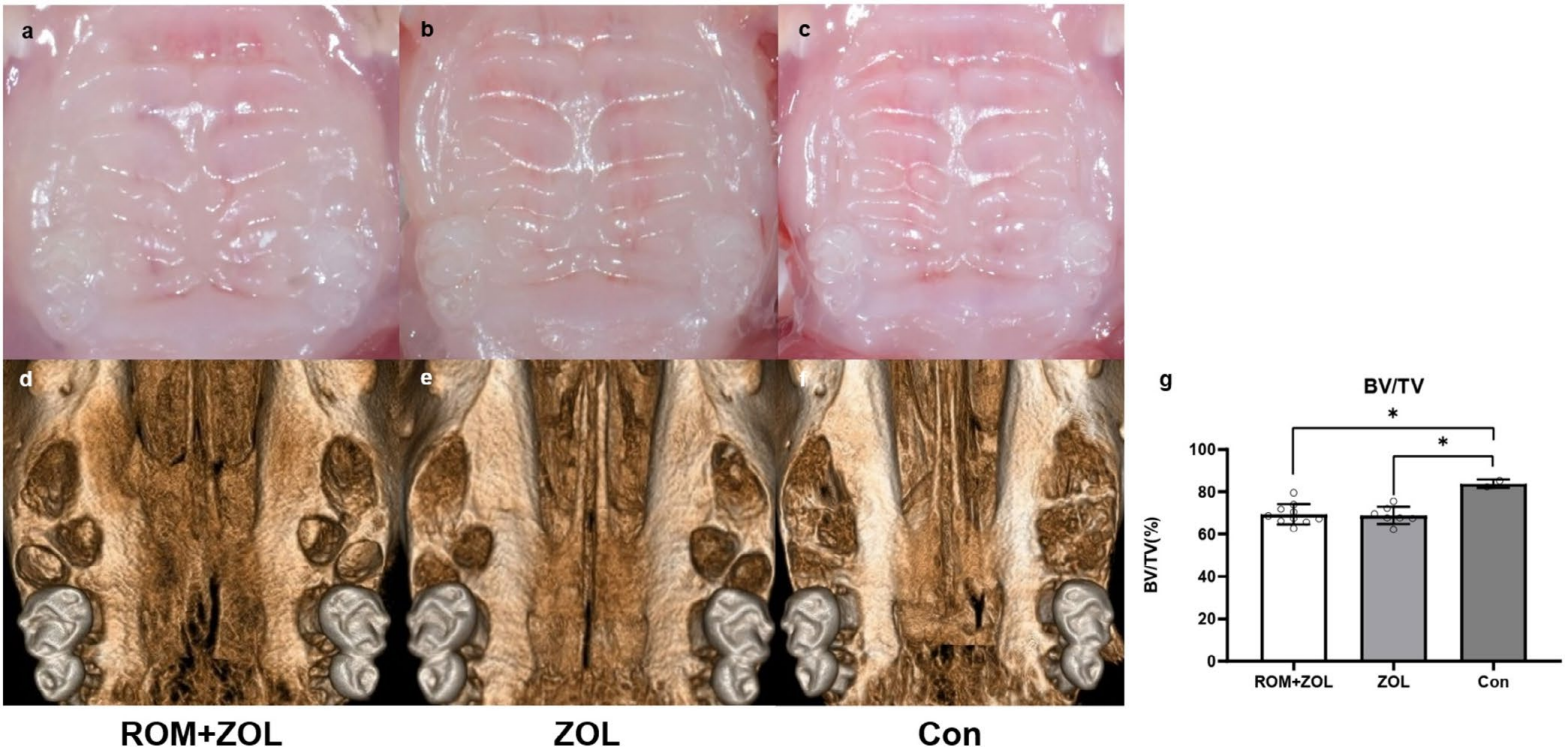
Analysis at 3 weeks after extraction. (a–c) Clinical images of each group. (d–f) micro‐computed tomography reconstruction images of each group. (g) Bone volume fraction of each group. *p < 0.05.

#### 3.1.2 Histomorphometric Analysis

##### 3.1.2.1 H&E Staining

The number of empty lacunae per unit area (number/mm^2^) was 50.32 ± 25.92 in the ROM+ZOL group, 23.22 ± 9.46 in the ZOL group, and 7.64 ± 5.86 in the Con group, with significant differences observed among all groups (Figure 3a–d).

**FIGURE 3:**
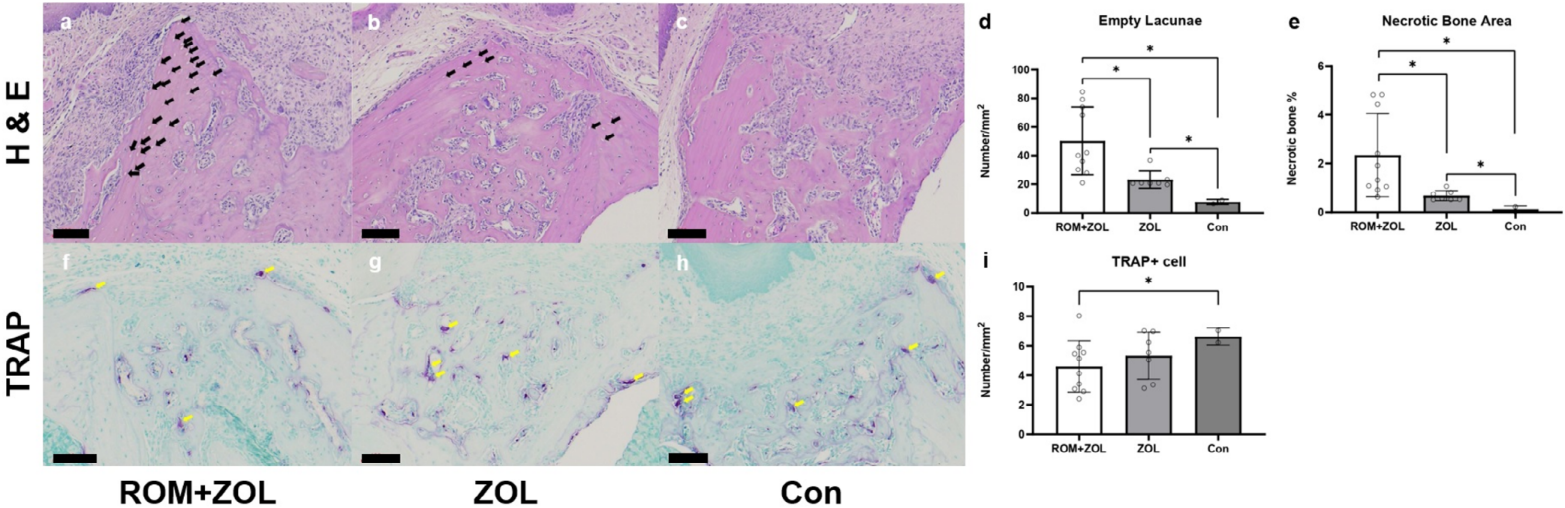
Histomorphometric analysis at 3 weeks after extraction. (a–c) Haematoxylin and eosin stained tissues at tooth extraction site (Black arrow: empty lacunae). (d) Quantification of empty lacunae per mm^2^. (e) Quantification of necrotic bone in percentage. (f–h) Tartrate‐resistant acid phosphatase staining (TRAP) stained tissues at tooth extraction site (Yellow arrow: TRAP+ cell). (i) Quantification of TRAP+ cell per mm^2^. *p < 0.05. Scale bars = 100 μm.

Similarly, the percentage of necrotic bone was 2.34 ± 1.93 in the ROM+ZOL group, 0.68 ± 0.55 in the ZOL group, and 0.12% ± 0.23% in the Con group, also showing significant differences among the groups (Figure 3e).

##### 3.1.2.2 TRAP Staining

The number of TRAP‐positive cells per unit area (number/mm^2^) was 4.60 ± 2.44 in the ROM+ZOL group, 5.33 ± 2.69 in the ZOL group, and 6.63 ± 2.70 in the Con group. A significant difference was observed only between the ROM+ZOL and Con groups (Figure 3f–i).

#### 3.1.3 Immunohistochemical Analysis

##### 3.1.3.1 RANKL, OPG, and RANKL/OPG Ratio

The proportion of RANKL‐positive staining per unit area was 2.81% ± 2.10% in the ROM+ZOL group, 1.55% ± 0.99% in the ZOL group, and 1.92% ± 1.80% in the Con group, with no significant differences among the groups. The percentage of OPG‐positive staining per unit area was 7.17% ± 4.56% in the ROM+ZOL group, 4.38% ± 1.56% in the ZOL group, and 8.62% ± 1.82% in the Con group. A significant difference was observed between the ZOL group and the other groups. The RANKL/OPG ratio was 0.44 ± 0.22 in the ROM+ZOL group, 0.38 ± 0.23 in the ZOL group, and 0.23 ± 0.32 in the Con group, with no significant differences among the groups (Figure 4a–i).

**FIGURE 4:**
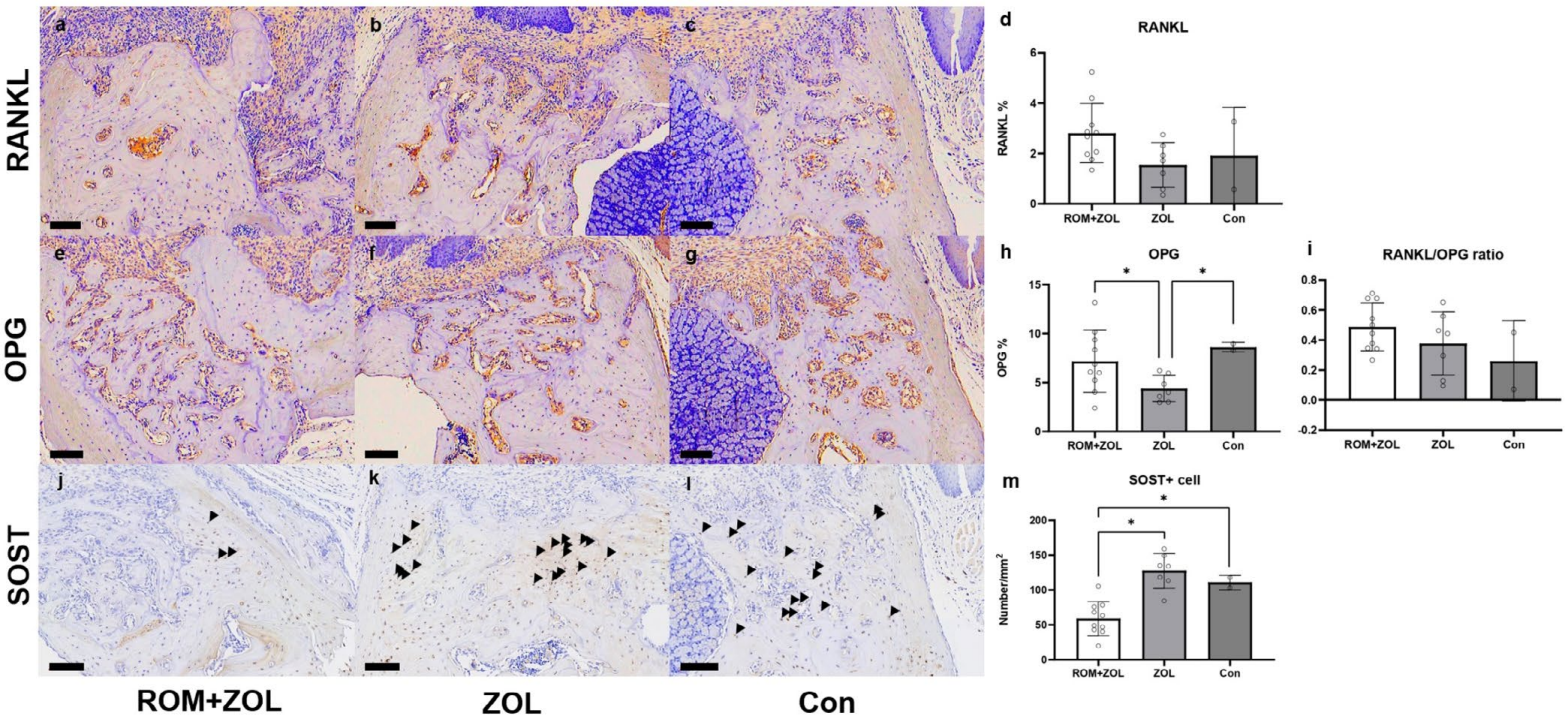
Immunohistochemical analysis at 3 weeks after extraction. (a–c) Receptor activator of nuclear factor‐κB ligand (RANKL) of each group. (d) Quantitative analysis of RANKL according to the group. (e–g) Osteoprotegerin (OPG) of each group. (h) Quantitative analysis of OPG according to the group. (i) Analysis of the RANKL/OPG ratio. (j–l) Sclerostin (SOST+) cell analysis of each group (Black triangle: SOST+ cell). (m) Quantitative analysis of SOST+ cell per mm^2^. *p < 0.05. Scale bars = 100 μm.

##### 3.1.3.2 SOST

The number of SOST‐positive cells per unit area (number/mm^2^) was 59.01 ± 31.86 in the ROM+ZOL group, 127.61 ± 30.14 in the ZOL group, and 110.67 ± 42.20 in the Con group. A significant difference was observed between the ROM+ZOL group and the other groups (Figure 4j–m).

### 3.2 In Vitro Cell Analysis

#### 3.2.1 Zoledronate and Romosozumab Inhibit Osteoclast Differentiation

F‐actin and TRAP staining are commonly used to assess osteoclast differentiation and function. TRAP staining detects TRAP, an enzymatic marker specific to osteoclasts, while F‐actin staining visualises the actin ring, a crucial cytoskeletal structure for bone resorption (Yang et al. 2019). To investigate the effects of zoledronate and romosozumab, osteoclast differentiation was induced and TRAP staining was performed to detect TRAP enzyme activity.

Our results demonstrated that osteoclasts treated with M‐CSF and sRANKL formed sealing zones, with TRAP activity. Treatment with zoledronate significantly reduced both the sealing zones and TRAP activity; however, some residual activity was still detectable. In contrast, romosozumab treatment resulted in a nearly complete loss of sealing zones and TRAP activity. Furthermore, when both drugs were administered together, sealing zones and TRAP activity were entirely absent (Figure 5a). Quantitative analysis confirmed a significant decrease in the number of osteoclasts maintaining podosome structures across all treatment groups (Figure 5b). Similarly, immunofluorescence analysis further demonstrated that actin rings were localised at the periphery of the differentiated osteoclasts. In the ZOL group, actin rings were partially preserved but markedly reduced. However, in the ROM and ROM+ZOL groups, actin rings were nearly undetectable (Figure 5c). This reduction in actin ring formation was statistically significant and followed a stepwise trend across the ZOL, ROM, and ROM+ZOL groups (Figure 5d). Our results suggest that zoledronate and romosozumab inhibit osteoclast differentiation by suppressing TRAP enzyme activity and disrupting actin ring formation, with a synergistic inhibitory effect observed when both agents are combined.

**FIGURE 5:**
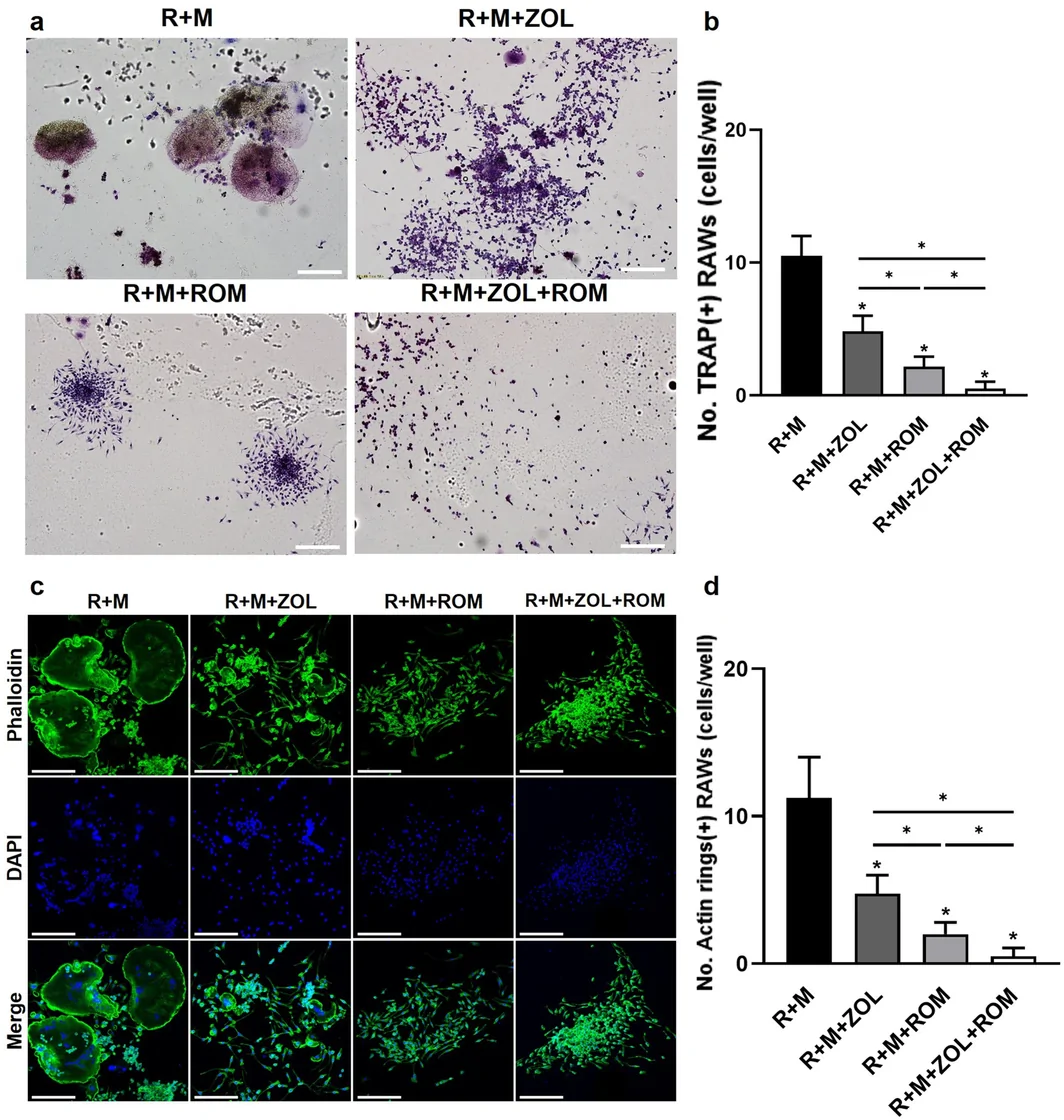
The effect of zoledronate and romosozumab on osteoclast differentiation. (a) Tartrate‐resistant acid phosphatase staining (TRAP) staining images (b) Quantitative analysis of TRAP‐positive multinucleated cells (MNCs) of RAW 264.7 cells treated with 25 μg/mL zoledronate and 6.45 mg/mL romosozumab. TRAP‐positive MNCs were defined as cells containing three or more nuclei. (c) Phalloidin staining images (d) quantitative analysis of phalloidin‐positive MNCs of RAW 264.7 cells treated with 25 μg/mL zoledronate and 6.45 mg/mL romosozumab. Actin ring formation was visualised around the cell periphery using phalloidin (green), while nuclei were counterstained with DAPI (blue). TRAP and immunofluorescence staining was performed after 3 days of drug treatment following stimulation with macrophage colony‐stimulating factor (M‐CSF) and RANKL. Scale bar = 20 μm. Data are presented as the means ± SEM of three independent experiments and statistical significance was analysed using one‐way ANOVA followed by Tukey’s HSD post hoc test, *p < 0.05. M, M‐CSF treatment; R, RANKL treatment; ROM, romosozumab; ZOL, zoledronate.

#### 3.2.2 Zoledronate and Romosozumab Suppress RANKL‐Induced Osteoclast Differentiation

To further evaluate the effects of zoledronate and romosozumab, we analysed protein expression following the differentiation of RAW 264.7 cells to examine their impact on RANKL‐induced osteoclast differentiation. The protein expression of RANK was significantly reduced in all treatment groups compared with the differentiation group, regardless of the drug administered (Figure 6a). However, no significant difference in RANK expression was observed between the ZOL and ROM groups (Figure 6b). The protein expression of NFATc1, a transcription factor regulating osteoclast differentiation, and cathepsin K, a key enzyme involved in bone resorption, was also suppressed in the ROM+ZOL group. Notably, their expression was significantly lower in the ROM group than in the ZOL group, with the combined ROM+ZOL treatment exhibiting the strongest inhibitory effect (Figure 6c,d). These results suggest that both zoledronate and romosozumab effectively suppress osteoclast differentiation and function, with their combined administration exhibiting a synergistic inhibitory effect.

**FIGURE 6:**
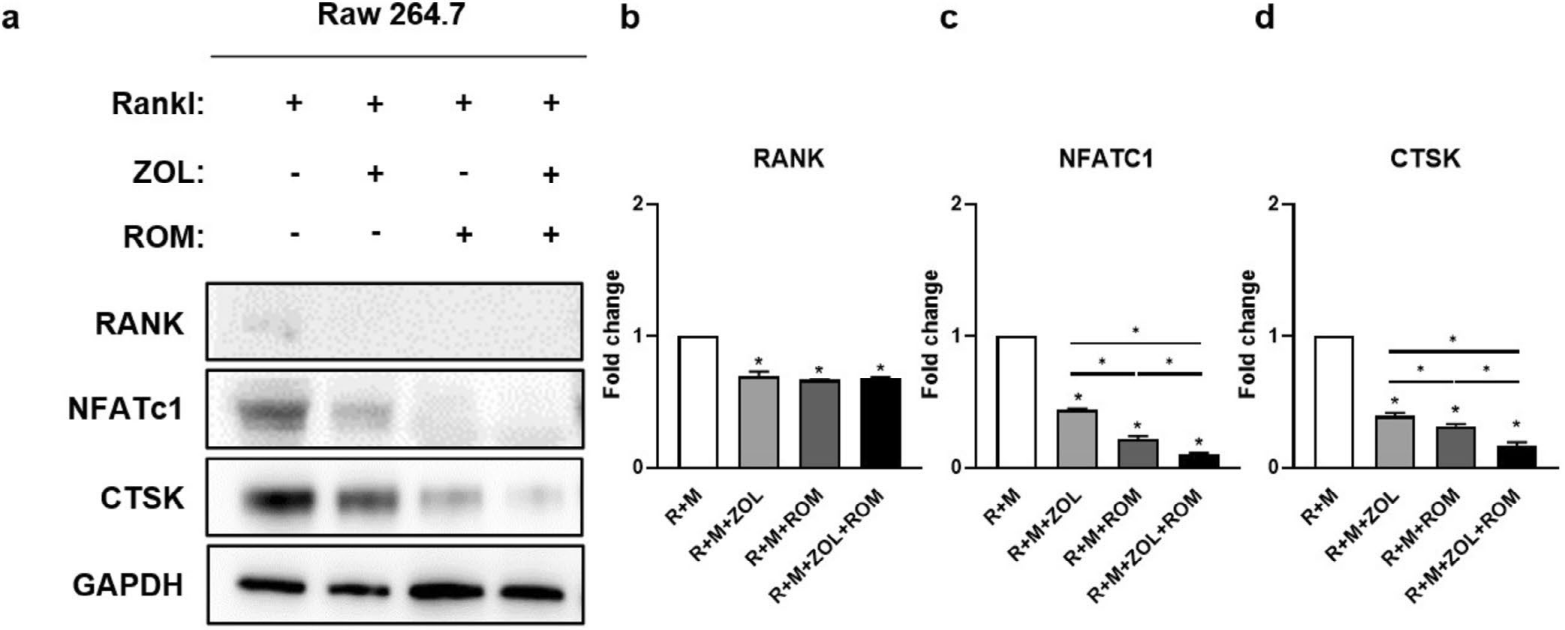
The effect of zoledronate and romosozumab on receptor activator of nuclear factor‐κB ligand (RANKL)‐induced osteoclast differentiation. (a) Western blot analysis and quantification of (b) RANK, (c) NFATc1 and (d) cathepsin K in RAW 264.7 cells treated with 25 μg/mL zoledronate and 6.45 mg/mL romosozumab. *GAPDH* was used as a loading control for normalisation. Data are presented as the means ± SEM of three independent experiments and statistical significance was analysed using one‐way ANOVA followed by Tukey’s HSD post hoc test, *p < 0.05.

## 4 Discussion

Various pharmacological agents are used in osteoporosis treatment, such as bisphosphonates, selective oestrogen receptor modulators, denosumab, parathyroid hormone (Tu et al. 2018), vitamin D, and calcium (Tu et al. 2018; Weaver et al. 2016). Among these, bisphosphonates and denosumab are widely employed as anti‐resorptive therapies, and numerous studies have reported an association between these treatments and MRONJ. Since the approval of romosozumab for osteoporosis treatment in 2019, its clinical use has increased, and cases of osteonecrosis of the jaw (ONJ) have also been reported in patients receiving this drug. However, no rodent studies have investigated the potential role of romosozumab in MRONJ models. In this study, we aimed to investigate whether the sequential administration of romosozumab followed by zoledronate could induce MRONJ in a murine model and to assess the inhibitory effects of zoledronate and romosozumab on osteoclast differentiation in vitro.

The study results showed that bone necrosis occurred in all groups treated with zoledronate, with the most severe necrosis observed in the group that received romosozumab before zoledronate. Micro‐CT revealed that the BV/TV ratio in the extraction sockets was significantly lower in both the ROM+ZOL and ZOL groups than in the Con group. These findings are consistent with those reported by Biguetti et al. (2019) and Soundia et al. (2021), who observed reduced BV/TV ratios in rodent models treated with zoledronate.

Additionally, the number of empty lacunae, a critical histological marker of MRONJ (Fondi and Franchi 2007; Sharma et al. 2013), was highest in the ROM+ZOL group, followed by the ZOL and Con groups. A similar trend was observed in the percentage of necrotic bone area, indicating that romosozumab exacerbates the necrotic effects induced by zoledronate. Romosozumab exerts its effects by binding to sclerostin and preventing it from inhibiting the Wnt signalling pathway, thereby enhancing bone formation through the promotion of osteoblast differentiation (Rauner et al. 2021). In parallel, it also inhibits bone resorption by reducing the RANKL/OPG ratio, resulting in dual osteoanabolic and anti‐resorptive actions. Immunohistochemical analysis revealed a significantly lower number of SOST‐positive cells in the ROM+ZOL group, confirming that romosozumab effectively suppressed sclerostin expression. Theoretically, such inhibition of sclerostin should reactivate the Wnt/β‐catenin pathway to promote bone healing. However, the severe osteonecrosis observed in this study implies a paradoxical outcome driven by the unique pharmacological profile of romosozumab. Unlike teriparatide, which functions primarily as an anabolic agent (Díez‐Pérez et al. 2019), romosozumab possesses a dual effect. Consequently, when zoledronate was administered in the presence of the anti‐resorptive activity of romosozumab, the combined action of these two drugs likely resulted in an aggravated suppression of osteoclasts. While this synergy might be advantageous for increasing bone mineral density, it appears detrimental in the context of MRONJ pathogenesis. These findings suggest that the synergistic blockade of osteoclast function severely compromised normal bone turnover and healing mechanisms, ultimately resulting in the most severe osteonecrosis.

To validate this hypothesis of aggravated suppression, we analysed osteoclast activity, a critical factor in MRONJ pathogenesis (Soundia et al. 2021; Yadegari et al. 2020). In the present study, TRAP staining demonstrated a trend toward reduced osteoclast numbers in the ROM+ZOL and ZOL groups compared to the Con group, with a significant decrease observed in the ROM+ZOL group. These findings are consistent with those of Gao et al. (2021), who also reported a reduction in TRAP‐positive cells following zoledronate administration in MRONJ models. Although statistically insignificant, the RANKL/OPG ratio exhibited a decreasing trend in the order of the ROM+ZOL, ZOL, and Con groups. Specifically, the ROM+ZOL group demonstrated the highest RANKL/OPG ratio. While romosozumab is generally known to decrease the RANKL/OPG ratio, the elevated ratio observed in this study appears to be driven by severe inflammation resulting from the extensive osteonecrosis induced by the concurrent application of zoledronate. In such inflammatory environments, immune cells are known to significantly upregulate RANKL expression (Chen et al. 2014). In the ZOL group, both RANKL and OPG levels were low, suggesting a state of reduced bone metabolism. The Con group exhibited the lowest ratio, which is presumed to reflect a return to a physiological homeostasis between RANKL and OPG following successful healing.

In this study, RAW 264.7 cells were differentiated into osteoclasts to evaluate the effects of zoledronate, romosozumab, and their combination by analysing key osteoclast markers. Overall, RANK expression was lower in the ZOL, ROM, and ROM+ZOL groups than in the Con group, although no significant differences were observed among the experimental groups. Conversely, NFATc1 and cathepsin K expression exhibited a decreasing trend across the treatment groups, with the highest levels in the ZOL group, followed by the ROM group and then in the ROM+ZOL group. These results indicate that although RANK expression, which is typically expressed at high levels in osteoclast precursors, was similarly suppressed across the treatment groups, osteoclast differentiation was progressively inhibited in the ZOL, ROM, and ROM+ZOL groups.

Assessments of actin ring formation and TRAP‐positive cell numbers, both indicators of osteoclast activity, revealed similar inhibitory trends across treatment groups. Zoledronate is known to induce osteoclast apoptosis, as confirmed in a previous study (Kellinsalmi et al. 2005). In contrast, romosozumab acts by inhibiting sclerostin, a protein primarily expressed by osteocytes, which leads to osteoblast activation and suppresses osteoclast activity. Some studies have also reported that sclerostin is expressed in osteoclast precursors, with its expression decreasing as these cells mature (Pederson et al. 2008). Based on this evidence, our results suggest that romosozumab inhibits osteoclast differentiation at the precursor stage.

The differences in osteoclast inhibition observed between the ZOL and ROM groups likely reflect their distinct mechanisms of action. Zoledronate primarily targets mature osteoclasts, inhibiting their bone‐resorptive function and inducing apoptosis rather than directly impairing their differentiation. In contrast, romosozumab is thought to act earlier in the differentiation process by inhibiting osteoclast precursor maturation (Pederson et al. 2008). The enhanced inhibition of osteoclast activity observed in the ROM+ZOL group can likely be attributed to the synergistic effects of these two drugs, combining zoledronate‐induced apoptosis in mature osteoclasts with romosozumab‐mediated suppression of precursor differentiation.

This study has some limitations that should be considered when interpreting the findings. While this study effectively demonstrated the development of osteonecrosis following sequential romosozumab and zoledronate treatment, the lack of data on romosozumab in combination with other anti‐resorptive agents, such as denosumab, represents a considerable limitation. This also includes the use of high doses of zoledronate, which were employed to robustly induce the MRONJ model in mice, and thus the results should be interpreted cautiously when extrapolated to a clinical patient setting. Additionally, the quantification of SOST, RANKL, and OPG was based on immunohistochemical analysis, which may be less accurate than molecular techniques such as reverse transcription‐polymerase chain reaction or serum‐level assessments.

In conclusion, the sequential administration of romosozumab and zoledronate resulted in severe bone necrosis, a reduction in the number of SOST‐positive cells, and suppression of osteoclast activity. These findings suggest a heightened risk of MRONJ when romosozumab treatment is combined with zoledronate, highlighting the need for caution in applying such treatment strategies in patients with osteoporosis. Moreover, based on recommendations by Shoback et al. (2020), who advocate for the use of anti‐resorptive agents following romosozumab in patients with low T‐scores, the potential development of MRONJ should be considered with the use of such treatment regimens. Further research is warranted to better understand the mechanisms underlying this risk and to develop effective strategies for the prevention and management of MRONJ in patients undergoing sequential therapy.

## Author Contributions

**Hae‐Seo Park:** investigation, formal analysis, visualization, writing – original draft. **Shihyun Kim:** investigation, visualization, formal analysis, writing – original draft. **Seo‐Yeong An:** investigation, formal analysis. **Reuben H. Kim:** writing – review and editing. **Jongho Choi:** investigation, methodology, supervision, writing – review and editing. **Moon‐Young Kim:** conceptualization, funding acquisition, methodology, supervision, writing – review and editing.

## Funding

National Research Foundation of Korea (NRF) grant funded by the Ministry of Science and ICT (RS‐2021‐NR061489).

## Ethics Statement

The study was approved by the Dankook University Institutional Animal Care and Use Committee (DKU‐23‐004), and all procedures adhered to the relevant regulations for animal experimentation.

## Consent

The authors have nothing to report.

## Conflicts of Interest

The authors declare no conflicts of interest.

## Data Availability

The data that support the findings of this study are available from the corresponding author upon reasonable request.
